# Frequency of maternal supplementation of energy and protein during late gestation modulates preweaning growth of their beef offspring

**DOI:** 10.1093/tas/txac110

**Published:** 2022-08-18

**Authors:** Vinicius Izquierdo, Marcelo Vedovatto, Elizabeth A Palmer, Rhaiza A Oliveira, Hiran M Silva, João M B Vendramini, Philipe Moriel

**Affiliations:** IFAS – Range Cattle Research and Education Center, University of Florida, Ona, FL 33865, USA; IFAS – Range Cattle Research and Education Center, University of Florida, Ona, FL 33865, USA; IFAS – Range Cattle Research and Education Center, University of Florida, Ona, FL 33865, USA; IFAS – Range Cattle Research and Education Center, University of Florida, Ona, FL 33865, USA; IFAS – Range Cattle Research and Education Center, University of Florida, Ona, FL 33865, USA; IFAS – Range Cattle Research and Education Center, University of Florida, Ona, FL 33865, USA; IFAS – Range Cattle Research and Education Center, University of Florida, Ona, FL 33865, USA

**Keywords:** beef cows, frequency, gestation, offspring, supplementation

## Abstract

This study evaluated the effects of decreasing the frequency of dried distillers grains (DDG) supplementation during third trimester of gestation on cow physiology and offspring preweaning growth. At 201 ± 7 d prepartum (day 0 of the study), 120 Brangus crossbred cows were stratified by body weight (BW = 543 ± 53 kg) and body condition score (BCS = 5.47 ± 0.73), and then assigned randomly to 1 of 20 bahiagrass (*Paspalum notatum*) pastures (six cows and 4.7 ha/pasture). Treatments were randomly assigned to pastures (five pastures/treatment) and consisted of cows offered no DDG supplementation (NOSUP) or precalving supplementation of DDG dry matter at 1 kg/cow daily (7×), 2.33 kg/cow every Monday, Wednesday, and Friday (3×), or 7 kg/cow every Monday (1×) from day 0 to 77. All cows assigned to DDG supplementation received the same total amount of DDG dry matter (77 kg/cow) from day 0 to 77. All cow-calf pairs were managed similarly from day 77 until calf weaning (day 342). Supplementation frequency did not impact (*P* ≥ 0.16) any forage or cow reproduction data. Cow BCS on days 77, 140, and 342 did not differ among 1×, 3×, and 7× cows (*P* ≥ 0.29) but all supplemented cows, regardless of supplementation frequency, had greater BCS on days 77, 140, and 342 compared to NOSUP cows (*P* ≤ 0.04). Cows offered 1× supplementation had greater plasma concentrations of IGF-1 on days 35 and 140 compared to NOSUP, 3× and 7× cows (*P* ≤ 0.04), whereas 3× and 7× cows had greater plasma concentrations of IGF-1 on day 35 compared to NOSUP cows (*P* ≤ 0.005). Average plasma concentrations of glucose did not differ among 1×, 3×, and 7× cows (*P* ≥ 0.44), but all supplemented cows had greater plasma concentrations of glucose compared to NOSUP cows (*P* ≤ 0.05). Birth BW of the first offspring did not differ between 3× and 7× calves (*P* = 0.54) but both groups were heavier at birth compared to NOSUP calves (*P* ≤ 0.05). On day 342, calves born from 7× cows were the heaviest (*P* ≤ 0.05), whereas calves born from 1× and 3× cows had similar BW (*P* = 0.97) but both groups were heavier compared to calves born from NOSUP cows (*P* ≤ 0.05). In summary, decreasing the frequency of DDG supplementation, from daily to one or three times weekly, during third trimester of gestation of beef cows did not impact cow BCS but altered maternal plasma concentrations of IGF-1 and glucose, leading to reduced offspring preweaning growth.

## Teaser

Decreasing the frequency of maternal supplementation of protein and energy during third trimester of gestation did not affect cow performance but altered their prepartum plasma concentrations of glucose and insulin-like growth factor 1 and reduced preweaning growth of their offspring. These data indicate that a more consistent supply of supplemental protein and energy during third trimester of gestation does not improve maternal performance but was required to optimize calf growth performance during the preweaning stage.

## INTRODUCTION

Decreasing the frequency of concentrate supplementation to beef cattle (from daily to three times weekly) reduced labor and feeding costs (Cooke et al., 2008) and led to variable effects on growth performance, either decreasing (Cooke et al., 2008; Moriel et al., 2012; Artioli et al., 2015) or not impacting their body weight (BW) gain (Moriel et al., 2016a, b; Silva et al., 2018). However, suboptimal immune function and reproductive performance were consistent outcomes to beef cattle receiving infrequent supplementation compared to daily concentrate supplementation (Cooke et al., 2008; Moriel et al., 2012, Artioli et al., 2015; Moriel et al., 2016b; Silva et al., 2018; Moriel et al., 2020a), which were associated with the greater fluctuations in daily intake of forage and nutrients. Infrequent supplementation altered synthesis and release of hormones and metabolites associated with energy and protein metabolism (Moriel et al., 2012; Artioli et al., 2015; Moriel et al., 2020a).

Nutritional status during third trimester of gestation of beef cows modulates placental environment, fetal development (Funston et al., 2012), and offspring postnatal performance (Palmer et al., 2020, 2022a, b). Reducing the frequency of concentrate supplementation during third trimester of gestation did not impact offspring birth BW (Klein et al., 2014) but was detrimental to offspring physiological stress following birth (Moriel et al., 2016a). Carryover effects of reduced maternal supplementation during late gestation on offspring preweaning growth was not detected (Moriel et al., 2016a), which could partially be attributed to the high-moisture content of the supplement utilized leading to slower supplement consumption (Arthington et al., 2004) and lessened daily fluctuations in nutrient intake and metabolism (Moriel et al., 2016a) compared to supplements with high dry matter concentration that are consumed relatively quick. We hypothesized that reducing the frequency of dried distillers grains (DDG) during third trimester of gestation of beef cows would not impact cow performance but would be detrimental to preweaning growth of their offspring due to greater fluctuations in hormones and metabolites during pregnancy compared to daily concentrate supplementation. Thus, our objectives were to evaluate the effects of decreasing the frequency of DDG supplementation during late gestation from daily to either once or three times weekly on cow physiology and preweaning growth of their beef offspring.

## MATERIALS AND METHODS

The study was conducted from September 2019 until July 2020 at the UF/IFAS Range Cattle Research & Education Center, Ona, FL (27° 26ʹ N and 82° 55ʹ W). All cow-calf pairs were cared following the protocol (#201910815) approved by the Institutional Animal Care and Use Committee from the University of Florida.

### Animals and Diets

At approximately 201 ± 7 d prepartum (day 0 of the study), 120 multiparous, Brangus crossbred beef cows (9 ± 3 yr of age) pregnant by natural breeding (10 Brangus bulls; 1 bull per 20 cows) were ranked by their initial BW (543 ± 53 kg) and body condition score (BCS = 5.47 ± 0.73), and then assigned randomly to 1 of 20 bahiagrass (*Paspalum notatum*) pastures (6 cows and 4.7 ha/pasture). Treatments were randomly assigned to pastures (5 pastures/treatment) and consisted of cows offered no precalving supplementation of DDG from day 0 to 77 (NOSUP) or precalving supplementation of DDG dry matter (DM) at 1 kg/cow daily (7×), 2.33 kg/cow every Monday, Wednesday, and Friday (3×), or 7 kg/cow every Monday (1×) from day 0 to 77. All cows assigned to DDG supplementation received the same total amount of DDG dry matter (77 kg/cow) to gain at least 0.5 unit of BCS (1 to 9 scale) from day 0 to 77 (Palmer et al., 2022a). Each respective DDG supplement amount was offered in open plastic feed bunks at 0800 h from day 0 to 77. Average weekly nutritional composition of DDG consisted of (DM basis): 89.1% DM, 33.1% crude protein (CP), 82.5% total digestible nutrients (TDN), 35.2% neutral detergent fiber, 15.2% acid detergent fiber, 0.04% Ca, 1.12% P, 0.33% Mg, 1.28% K, 0.26%Na, 0.69% S, 88 mg/kg Fe, 77 mg/kg Zn, 7 mg/kg Cu, 17 mg/kg Mn, and 1.7 mg/kg Mo. A commercial complete trace mineral and vitamin mixture was delivered once weekly for a target daily consumption of 56 g/cow throughout the entire study (16.8% Ca, 1.0% Mg, 20.7% NaCl, 4.0% P, 60 mg/kg Co, 1,750 mg/kg Cu, 350 mg/kg I, 60 mg/kg Se, and 5,000 mg/kg Zn; Vigortone, Brookville, OH).

On day 77, each cow was randomly distributed to 1 of 10 groups (12 cows/group; all treatments equally represented in each group) and then each group was allocated into 1 of 10 bahiagrass pastures (9.4 ha/pasture). From day 110 to 231, each group was offered stargrass (*Cynodon nlemfuensis*) hay ad libitum and provided a commercial sugarcane molasses and urea supplement at 12.4 kg of DM per cow weekly (82.4% DM; 22% CP and 75% TDN; Westway Feed Products LLC, Clewiston, FL). Molasses and urea supplement was provided at 0800 h every Monday and Thursday in open plastic tanks placed 1 m above the ground to avoid calf consumption of supplement (Palmer et al., 2020, 2022a). All groups were checked daily for calving starting on day 77. Calves were allowed to consume maternal colostrum and then were weighed, tagged, and castrated (if male) within 24 h following birth. Brangus crossbred bulls were placed with cows (one bull/group) and rotated among all groups monthly from day 140 to 231. On day 231, cows and their calves received an oral anthelmintic treatment (Safe-Guard; Merck Animal Health, Madison, NJ) and calves were vaccinated against *Clostridium* (Ultrabac 8, Zoetis, Parsippany, NJ) and respiratory pathogens (Bovi Shield Gold One Shot, Zoetis), following the recommended manufacture dosage. All calves were weaned on day 342.

### Sample and Data Collection

Herbage mass of bahiagrass pastures was determined on days 0, 35, and 77 following the double sampling technique described by Gonzalez et al. (1990). Herbage allowance was calculated on days 0, 35, and 77 according to Sollenberger et al. (2005) by dividing the herbage mass of each pasture by the respective total cow and calf BW on the respective pasture. Hand-plucked samples of each pasture were obtained on days 0, 35, and 77. All forage samples were placed in a forced-air oven and dried at 55 °C for 72 h, and then ground to pass a 4-mm stainless steel screen using a Thomas-Wiley Laboratory Mill (Model 4; Thomas Scientific, Swedesboro, NJ). All processed forage samples were submitted to the University of Florida Forage Evaluation Support Laboratory to determine the concentrations of CP using the micro-Kjeldahl technique (Gallaher et al., 1975) and in vitro digestible organic matter (IVDOM; Moore and Mott, 1974).

Cow unshrunk BW and BCS (1 to 9 scale, according to Wagner et al., 1988) were recorded on days 0, 35, 77, 140 (start of the breeding season), and 342 (weaning). Feed and water withdraw was not implemented to avoid disturbing feeding behavior and any resulting physiological stress which could impact cow plasma data and offspring growth following birth (Marques et al., 2012). Approximately 10 mL of blood samples were collected from three cows/pasture (randomly selected on day 0) on days 0, 35, 77, and 140. Cow blood samples were collected via jugular venipuncture into commercial tubes (Vacutainer, Becton Dickinson, Franklin Lakes, NJ) containing 158 USP of sodium-heparin between 1100 and 1200 h (approximately 3 to 4 h after DDG supplementation) to correspond with the greatest concentrations and release of ruminal fermentation end products (Moriel et al., 2016b). Blood samples were collected from three male and three female calves/pasture, within 12 h after birth but after colostrum consumption, into commercial tubes containing no additive (Vacutainer, Becton Dickson). Blood samples were placed on ice immediately after collection, and then centrifuged at 4 °C and 1,200×*g* for 25 min. Plasma was then harvested and kept frozen at −20 °C until further laboratory analysis. Cow plasma samples were obtained to determine the concentrations of glucose, insulin-like growth factor-1 and insulin-like growth factor-2 (IGF-1 and IGF-2), and non-esterified fatty acids (NEFA). Plasma concentrations of IGF-2 of cows were only assessed during the prepartum period (days 0, 35, and 77) as IGF-2 increases with gestation length (Kubota et al., 1992). Serum samples of calves were obtained to assess the serum concentrations of immunoglobulin G (IgG).

Percentage of pregnant cows on day 276 (45 d after bull removal) was performed via rectal palpation by a trained veterinarian. Unshrunk calf BW was recorded individually within 12 h after birth and then on days 140 and 342. Additional preweaning data of cows and calves from day 141 to 342 and post-weaning data of calves were not obtained due to the onset of the coronavirus disease (COVID-19) pandemic in February 2020 and the subsequent university mandate to cease research data collection during spring and summer 2020. Only normal operating procedures (e.g., pregnancy percentage on day 276 and calf weaning and BW collection on day 342) were executed following the guidelines established by the Centers for Disease Control and Prevention (Atlanta, GA, USA) and upon authorization by the University of Florida - IFAS Research office.

### Laboratory Procedures

Commercial ELISA kits validated for bovine samples were utilized to determine the cow plasma concentrations of IGF-1 (SG100; R&D Systems, Inc., Minneapolis, MN; Moriel et al., 2012) and IGF-2 (LS-F51244; LifeSpan BioSciences, Inc., Seattle, WA; Palmer et al., 2020) and calf serum concentrations of IgG (E11-118; Bethyl Laboratories, Inc., Montgomery, TX).-Commercial bovine quantitative colorimetric kits were utilized to assess the plasma concentrations of glucose (#G7521; Pointe Scientific, Inc., Canton, MI) and NEFA (HR Series NEFA-2; Wako Pure Chemical Industries, Ltd. USA, Richmond, VA; Pescara et al., 2010). Intra-assay and inter-assay CV for assays for concentrations of glucose, IGF-1, IGF-2, NEFA, and IgG were 2.23 and 2.86, 2.96 and 3.12, 4.77 and 4.99, 3.46 and 4.11 and 3.23 and 2.55%, respectively.

### Statistical Analyses

Except for binary data, all data collected herein were analyzed as a complete randomized study using pasture as the experimental unit and MIXED procedure of SAS (SAS Institute Inc., Cary, NC, version 9.4). Pasture(supplementation frequency) and cow(pasture) or calf(pasture) were included in the model as random effects in all statistical analyses of cow and calf performance. Pasture(supplementation frequency) was the only random effect included in the model in the statistical analyses of herbage mass and allowance. Calf BW at birth, serum concentrations of IgG at birth, and calf preweaning average daily gain (ADG) were tested for fixed effects of supplement frequency. Cow BW and BCS, calf BW, cow plasma data, and all forage data were analyzed as repeated measures and evaluated for fixed effects of supplement frequency, day of the study, and supplement frequency × day of the study. Cow(pasture), calf(pasture), and pasture were included as subjects in the repeated measures analyses of cow, calf, and forage data, respectively. The compound symmetry covariance structure was selected for all analyses of repeated measures because it generated the lowest Akaike information criterion. Cow BCS and BW on day 0 did not differ among treatments (*P* ≥ 0.72) but were included as covariates (*P* < 0.0001) in the statistical analyses of cow BCS and BW, respectively. Effects of calf sex, sire, calving date, and birth BW were included as covariates in the model for all calf performance variables, but removed from the model when *P* ≥ 0.10. First offspring BW and ADG were covariate-adjusted to calf sex and calving date (*P* ≤ 0.0008). Percentage of cows that calved and male calves at birth (first and second offspring) and percentage of pregnant cows on day 276 were tested for fixed effects of supplement frequency using pasture as the experimental unit and GLIMMIX procedure of SAS. Results were reported as least-square means and means were separated using PDIFF when a significant *F*-test was detected. Significance was set at *P* ≤ 0.05 and tendencies when *P* > 0.05 and ≤ 0.10.

## RESULTS

Effects of day of the study, but not supplementation frequency × day of the study and supplementation frequency (*P* ≥ 0.16), were detected for herbage mass, herbage allowance, IVOMD, and CP (*P* < 0.0001; Table 1). Herbage mass and allowance were greatest on day 0 (*P* < 0.0001), decreased on day 35 (*P* < 0.0001), and did not differ between days 35 and 77 (*P* ≥ 0.17; Table 1). Forage IVOMD and CP gradually decreased from day 0 to 35 (*P* ≤ 0.05; Table 1).

**Table 1.** Herbage mass, herbage allowance, IVDOM and CP of bahiagrass pastures provided to beef cows from day 0 to 77 (six cows and 4.7 ha/pasture)^1^

Item	Day of the study			SEM	*P*-value
	0	35	77		Day
Herbage mass, kg of DM/ha	6,259^b^	4,793^a^	4,846^a^	135.9	<0.0001
Herbage allowance, kg of DM/kg of BW	1.95^b^	1.38^a^	1.47^a^	0.044	<0.0001
IVDOM, %	44.9^a^	46.5^b^	36.7^c^	0.62	<0.0001
CP, % of DM	10.3^a^	9.1^b^	9.8^c^	0.12	<0.0001

Within a row, means without a common superscript differ (*P* ≤ 0.05).

Herbage mass and allowance were assessed on days 0, 35, and 77, as described by Gonzalez et al. (1990) and Sollenberger et al. (2005), respectively.

Treatments consisted of no supplementation of DDG from day 0 to 77 (NOSUP) or supplementation of DDG DM at 1 kg/cow daily (7×), 2.33 kg/cow every Monday, Wednesday, and Friday (3×), or 7 kg/cow every Monday from day 0 to 77 (1×; five pastures/treatment). Treatments were provided to cows from day 0 to 77 (from 201 ± 7 d prepartum until 279 ± 7 d prepartum) leading to a total of 77 kg/cow of DDG dry matter for 1×, 3×, and 7× cows. On day 77, each cow was randomly assigned to 1 of 10 groups (12 cows/group; all treatments equally represented in each group). Thereafter, each group was assigned two 4.7-ha bahiagrass pastures and rotated between pastures weekly until day 342. From day 110 to 231, all cow-calf pairs were offered stargrass hay ad libitum and supplemented with sugarcane molasses + urea (12.4 kg of DM per cow weekly, which was divided by 2 and offered every Monday and Thursday).

Effects of supplementation frequency × day of the study were detected for cow BCS (*P* = 0.002) but not cow BW (*P* = 0.42; Table 2). Cow BCS on day 0 and 35 did not differ among treatments (*P* ≥ 0.46). Cow BCS on day 77, 140, and 342 did not differ among 1×, 3×, and 7× cows (*P* ≥ 0.29) but all supplemented cows, regardless of supplementation frequency, had greater BCS on day 77, 140, and 342 compared to NOSUP cows (*P* ≤ 0.04; Table 2). Cow BCS change from day 0 to 35 and day 140 to 342 did not differ among treatments (*P* ≥ 0.64). Cow BCS change differed among treatments from day 35 to 77 (*P* = 0.01) and tended to differ among treatments from day 77 to 140 (*P* = 0.07; Table 2). Cow BCS change from day 35 to 77 did not differ among 1×, 3×, and 7× cows (*P* ≥ 0.50), whereas NOSUP cows lost BCS compared to all supplemented groups, regardless of supplementation frequency (*P* ≤ 0.02; Table 2). Cow BCS change from day 77 to 140 did not differ among 1×, 3×, and 7× cows (*P* ≥ 0.65) but all supplemented cows, regardless of supplementation frequency, lost BCS compared to NOSUP cows (*P* ≤ 0.05; Table 2). Cow BW change from day 0 to 35 tended to differ among treatments (*P* = 0.09; Table 2), which did not differ among 1×, 3×, and 7× cows (*P* ≥ 0.71) but all supplemented cows, regardless of supplementation frequency, had greater BW change from day 0 to 35 compared to NOSUP cows (*P* ≤ 0.05; Table 2). Cow BW change from day 35 to 77, 77 to 140, and 140 to 342 did not differ among treatments (*P* ≥ 0.57).

**Table 2.** BCS, BW, and BCS and BW change of beef cows grazing bahiagrass pastures (six cows and 4.7 ha/pasture) and assigned to receive no supplementation of DDG from day 0 to 77 (NOSUP) or supplementation of DDG dry matter at 1 kg/cow daily (7×), 2.33 kg/cow every Monday, Wednesday, and Friday (3×), or 7 kg/cow every Monday from day 0 to 77 (1×; five pastures/treatment)

Item	Supplementation frequency^1^				SEM	*P*-value	
	NOSUP	1×	3×	7×		Supplementation frequency	Supplementation freq. × day
BCS^2^							
Day 0	5.46^a^	5.47^a^	5.47^a^	5.48^a^	0.092	0.0001	0.002
Day 35	5.26^a^	5.23^a^	5.35^a^	5.46^a^	0.092		
Day 77	4.75^a^	5.34^b^	5.36^b^	5.45^b^	0.092		
Day 140	4.76^a^	5.02^b^	5.05^b^	5.10^b^	0.092		
Day 342	5.04^a^	5.46^b^	5.38^b^	5.60^b^	0.092		
BCS change							
Day 0–35	−0.21	−0.24	−0.10	−0.14	0.124	0.85	-
Day 35–77	−0.51^a^	0.11^b^	−0.01^b^	0.13^b^	0.136	0.01	-
Day 77–140	0.01^b^	−0.32^a^	−0.28^a^	−0.35^a^	0.108	0.07	-
Day 140–342	0.28	0.43	0.32	0.49	0.129	0.64	-
BW^2^, kg							
Day 0	543	541	544	544	4.6	0.13	0.42
Day 35	570	582	585	584	4.6		
Day 77	569	577	576	569	4.6		
Day 140	516	523	520	522	4.6		
Day 342	542	555	548	560	4.6		
BW change, kg							
Day 0–35	28^a^	40^b^	42^b^	40^b^	4.2	0.09	-
Day 35–77	−1	−5	−9	−15	7.5	0.63	-
Day 77–140	−54	−56	−53	−47	6.5	0.74	-
Day 140–342	27	33	28	37	5.9	0.57	-

Within a row, means without a common superscript differ (*P* ≤ 0.05).

Treatments were provided to cows from day 0 to 77 (from 201 ± 7 d prepartum until 279 ± 7 d prepartum) leading to a total of 77 kg/cow of DDG dry matter for 1×, 3×, and 7× cows. On day 77, each cow was randomly assigned to 1 of 10 groups (12 cows/group; all treatments equally represented in each group) and randomly allocated into one 9.4-ha bahiagrass pasture. Each group was offered stargrass hay ad libitum and supplemented with sugarcane molasses and urea (12.4 kg of DM per cow weekly) from day 110 to 231. Brangus crossbred bulls were placed with cows (1 bull/group) from day 140 to 231.

Cow BCS and BW on day 0 did not differ among treatments (*P* ≥ 0.72) but were included as covariate (*P* < 0.0001) in the statistical analyses of cow BCS and BW.

Calf sex and age did not impact calf birth BW of first and second offspring (*P* ≥ 0.15) and were removed from the model. Effects of supplementation frequency tended to be detected for birth BW of the first offspring (*P* = 0.08), which did not differ between 3× and 7× calves (*P* = 0.54) but both groups were heavier at birth compared to NOSUP calves (*P* ≤ 0.05), whereas 1× calves were intermediate (*P* ≥ 0.18; Table 3). Effects of supplementation frequency were not detected for percentage of pregnant cows on day 276 (*P* = 0.39) and calving percentage, calving date, and percentage of male calves at birth of first and second offspring (*P* ≥ 0.15; Table 3).

**Table 3.** Reproductive performance of beef cows grazing bahiagrass pastures (six cows and 4.7 ha/pasture) and assigned to receive no supplementation of DDG from day 0 to 77 (NOSUP) or supplementation of DDG dry matter at 1 kg/cow daily (7×), 2.33 kg/cow every Monday, Wednesday, and Friday (3×), or 7 kg/cow every Monday from day 0 to 77 (1×; five pastures/treatment)

Item	Supplementation frequency^1^				SEM	*P*-value
	NOSUP	1×	3×	7×		Supplementation frequency
First offspring						
Calving percentage, % of total cows	93.3	86.7	96.7	100	5.29	0.15
Calving date, day of the study	86	76	81	80	6.7	0.75
Calf birth BW^2^, kg	33.2^a^	35.4^ab^	37.1^b^	36.1^b^	1.16	0.08
Male calves, % of total calves	55.2	53.6	44.8	53.3	9.41	0.86
Second offspring						
Pregnant day 276, % of total cows	93.3	81.5	85.7	93.3	5.91	0.39
Calving percentage, % of total cows	80.0	79.3	79.3	80.0	7.47	0.99
Calving date, day of the study	451	451	449	445	3.9	0.64
Calf birth BW^2^, kg	36.1	35.5	36.1	36.3	1.49	0.98
Male calves, % of total calves	40.0	45.5	50.0	43.5	10.8	0.92

Within a row, means without a common superscript differ (*P* ≤ 0.05).

Treatments were provided to cows from day 0 to 77 (from 201 ± 7 d prepartum until 279 ± 7 d prepartum) leading to a total of 77 kg/cow of DDG dry matter for 1×, 3×, and 7× cows. On day 77, each cow was randomly assigned to 1 of 10 groups (12 cows/group; all treatments equally represented in each group) and randomly allocated into one 9.4-ha bahiagrass pasture. Brangus crossbred bulls were placed with cows (1 bull/group) on day 140 and bulls were rotated among groups every 14 d until day 231. Pregnancy diagnosis was performed via rectal palpation by a trained veterinarian on day 276. All calves were weaned on day 342. All cows were managed similarly from day 77 until final data collection of second offspring birth date and birth body weight.

Individual calf birth BW was obtained within 12 h after birth and was not covariate-adjusted for calf sex or age (*P* ≥ 0.15).

**Figure 1.**
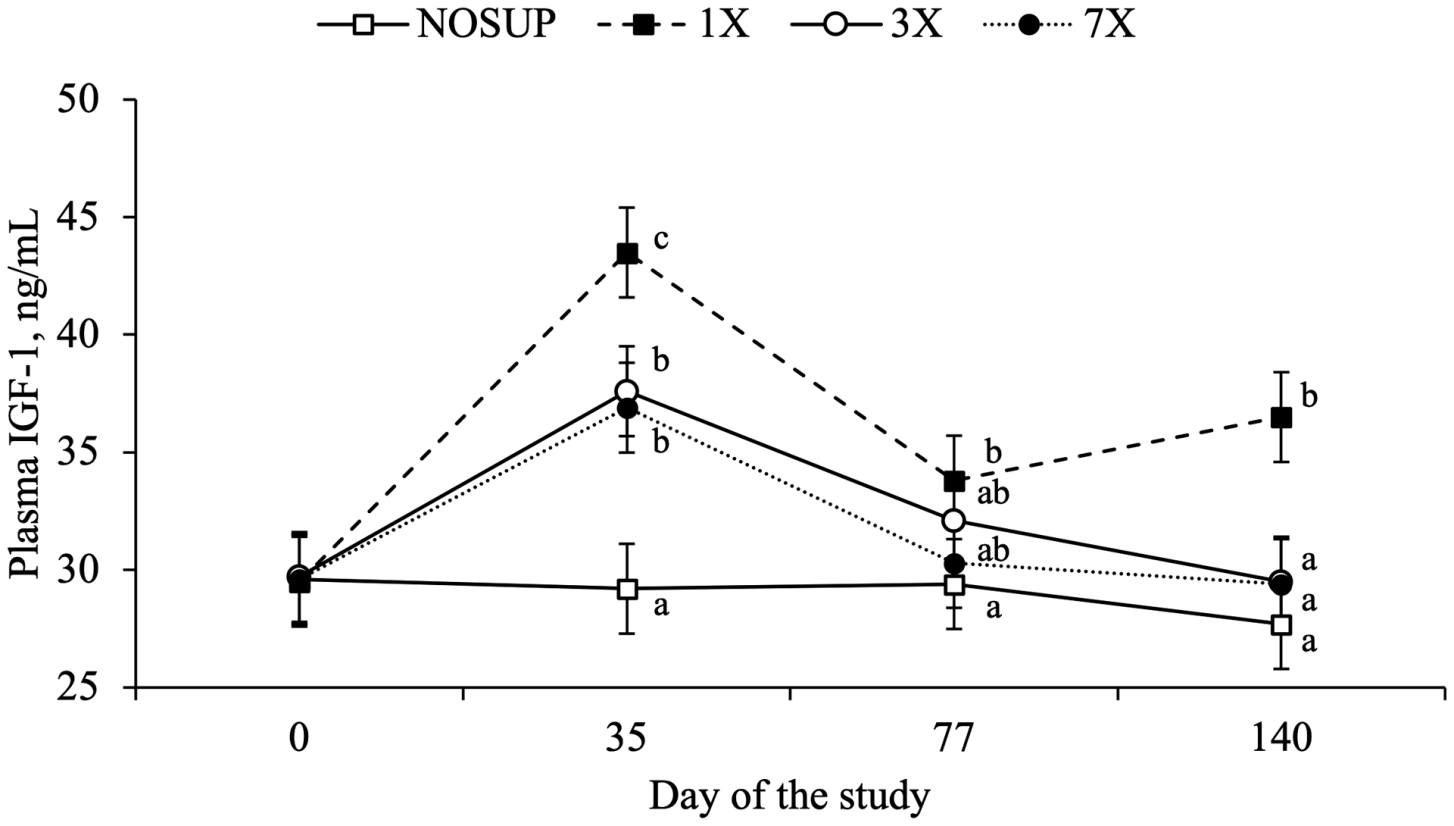
Plasma concentrations of IGF-1 of beef cows grazing bahiagrass pastures (six cows and 4.7 ha/pasture) and assigned to receive no supplementation of DDG from day 0 to 77 (NOSUP) or supplementation of DDG dry matter at 1 kg/cow daily (7×), 2.33 kg/cow every Monday, Wednesday, and Friday (3×), or 7 kg/cow every Monday from day 0 to 77 (1×; 5 pastures/treatment). Treatments were provided to cows from 201 ± 7 d prepartum until 279 ± 7 d prepartum leading to a total of 77 kg/cow of DDG dry matter for 1×, 3×, and 7× cows. On day 77, each cow was randomly assigned to 1 of 10 groups (12 cows/group; all treatments equally represented in each group) and randomly allocated into one 9.4-ha bahiagrass pasture. Each group was offered stargrass hay ad libitum and supplemented with sugarcane molasses and urea (12.4 kg of DM per cow weekly) from day 110 to 231. Effects of treatment × day of the study were detected for plasma concentrations of IGF-1 of beef cows. Plasma concentrations of IGF-1 on day 0 did not differ among treatments (*P* ≥ 0.93) but were included in the model as covariate (*P* = 0.0001). ^a–c^Within day of the study, means without a common letter differ (*P* ≤ 0.05).

Effects of supplementation frequency × day of the study were detected for cow plasma concentrations of IGF-1 (*P* = 0.02; Figure 1). Cows offered 1× supplementation had greater plasma concentrations of IGF-1 on days 35 and 140 compared to NOSUP, 3× and 7× cows (*P* ≤ 0.04) and greater plasma concentrations of IGF-1 on day 77 compared to NOSUP cows (*P* = 0.05; Figure 1). Plasma concentrations of IGF-1 on day 35 were greater for 3× and 7× cows compared to NOSUP cows (*P* ≤ 0.005) and did not differ among 3×, 7×, and NOSUP cows on days 77 and 140 (*P* ≥ 0.35; Figure 1). Effects of supplementation frequency, but not supplementation frequency × day of the study (*P* = 0.64), tended to be detected for cow plasma concentrations of glucose (*P* = 0.08; Table 4). Average plasma concentrations of glucose did not differ among 1×, 3×, and 7× cows (*P* ≥ 0.44), but all supplemented cows had greater plasma concentrations of glucose compared to NOSUP cows (*P* ≤ 0.05; Table 4). Effects of supplementation frequency × day of the study and supplementation frequency were not detected for cow plasma concentrations of IGF-2 and NEFA (*P* ≥ 0.39; Table 4). Effects of supplementation frequency were not detected for serum concentrations of IgG and plasma concentrations of haptoglobin of the first offspring at birth (*P* ≥ 0.37; Table 4).

**Table 4.** Average plasma concentrations of glucose, IGF-2, and NEFA of beef cows and serum concentrations of IgG and plasma concentrations of haptoglobin of their first offspring. Cows were assigned to receive no supplementation of DDG from day 0 to 77 (NOSUP) or supplementation of DDG dry matter at 1 kg/cow daily (7×), 2.33 kg/cow every Monday, Wednesday, and Friday (3×), or 7 kg/cow every Monday from day 0 to 77 (1×; five pastures/treatment)

Item^2^	Supplementation frequency^1^				SEM	*P*-value		
	NOSUP	1×	3×	7×		Supplementation frequency	Day	Supplementation freq. × day
Cows								
Plasma glucose, mg/dL	64.4^a^	67.9^b^	66.7^b^	67.3^b^	1.00	0.08	<0.0001	0.64
Plasma IGF-2, ng/mL	2,727	1,905	3,014	2,745	483.9	0.39	0.003	0.43
Plasma NEFA, mEq/L	0.66	0.62	0.68	0.64	0.033	0.61	<0.0001	0.59
First offspring								
Serum IgG at birth, mg/mL	44.3	46.9	58.4	47.6	8.68	0.65	-	-
Plasma haptoglobin at birth, mg/mL	0.18	0.18	0.17	0.15	0.011	0.37	-	-

Within a row, means without a common superscript differ (*P* ≤ 0.05).

Treatments were provided to cows from day 0 to 77 (from 201 ± 7 d prepartum until 279 ± 7 d prepartum) leading to a total of 77 kg/cow of DDG dry matter for 1×, 3×, and 7× cows. On day 77, each cow was randomly assigned to 1 of 10 groups (12 cows/group; all treatments equally represented in each group) and randomly allocated into one 9.4-ha bahiagrass pasture. Each group was offered stargrass hay ad libitum and supplemented with sugarcane molasses and urea (12.4 kg of DM per cow weekly) from day 110 to 231.

Plasma concentrations of glucose, IGF-2, and NEFA of cows were covariate-adjusted (*P* < 0.0001) for the respective plasma data obtained on day 0. Blood samples of cows were collected from three cows/pasture on days 0, 35, 77, and 140. Blood samples of the first offspring were collected from three steers and three heifers/pasture within 12 h after birth but after colostrum consumption.

Effects of supplementation frequency × day of the study were detected for BW of first offspring (*P* = 0.02; Table 5). On day 140, calves born from 7× cows were heavier compared to calves born from NOSUP and 1× cows (*P* ≤ 0.05), whereas calves born from 3× cows were intermediate (*P* ≥ 0.25; Table 5). On day 342, calves born from 7× cows were the heaviest (*P* ≤ 0.05), whereas calves born from 1× and 3× cows had similar BW (*P* = 0.97) but both groups were heavier compared to calves born from NOSUP cows (*P* ≤ 0.05; Table 5). Calf ADG from birth to day 140 did not differ among treatments (*P* = 0.16), whereas calf ADG from day 140 to 342 and birth to day 342 tended to differ among treatments (*P* ≤ 0.10; Table 5). Calves born from 7× cows had greater ADG from day 140 to 342 (*P* = 0.05) and tended to have greater ADG from birth to day 342 (*P* = 0.06) compared to calves born from NOSUP cows, whereas ADG from day 140 to 342 and birth to day 342 of calves born from 1× and 3× cows were intermediate and did not differ compared to 1× and 7× cows (*P* ≥ 0.36; Table 5).

**Table 5.** BW and ADG of first offspring born to beef cows grazing bahiagrass pastures (six cows and 4.7 ha/pasture) and assigned to receive no supplementation of DDG from day 0 to 77 (NOSUP) or supplementation of DDG dry matter at 1 kg/cow daily (7×), 2.33 kg/cow every Monday, Wednesday, and Friday (3×), or 7 kg/cow every Monday from day 0 to 77 (1×; five pastures/treatment)

Item	Supplementation frequency^1^				SEM	*P*-value	
	NOSUP	1×	3×	7×		Supplementation frequency	Supplementation freq. × day
First offspring BW^2^, kg							
Day 140	83^a^	85^a^	89^ab^	93^b^	3.4	0.14	0.05
Day 342	253^a^	261^b^	261^b^	269^c^	3.4		
First offspring ADG^2^, kg/d							
Birth until day 140	0.92	1.02	0.91	0.94	0.037	0.16	-
Day 140 to 342	0.58^a^	0.61^ab^	0.61^ab^	0.62^b^	0.012	0.09	-
Birth until day 342	0.77^a^	0.79^ab^	0.78^ab^	0.80^b^	0.019	0.10	-

Within a row, means without a common superscript differ (*P* ≤ 0.05).

Treatments were provided to cows from day 0 to 77 (from 201 ± 7 d prepartum until 279 ± 7 d prepartum) leading to a total of 77 kg/cow of DDG dry matter for 1×, 3×, and 7× cows. On day 77, each cow was randomly assigned to 1 of 10 groups (12 cows/group; all treatments equally represented in each group) and randomly allocated into one 9.4-ha bahiagrass pasture. Each group was offered stargrass hay ad libitum and supplemented with sugarcane molasses and urea (12.4 kg of DM per cow weekly) from day 110 to 231. Brangus crossbred bulls were placed with cows (one bull/group) from day 140 to 231. All calves were weaned on day 342.

Covariate-adjusted to calf sex and calving date (*P* ≤ 0.0008).

## DISCUSSION

Decreasing the frequency of concentrate supplementation leads to fluctuation in daily forage intake (primarily in supplemental concentrate with high- vs. low-starch concentrations), which decreased on days that concentrate supplementation was offered and increased (at different magnitudes) on days when concentrate supplementation was not offered, leading to either similar or reduced total weekly amount of forage consumed (Moriel et al., 2012; Artioli et al., 2015; Moriel et al., 2016a, b; Silva et al., 2018). Herbage allowance available to cows from day 0 to 77 did not differ among treatments, suggesting that weekly forage intake also did not differ among treatments. Nonetheless, herbage allowance was always above the minimum threshold (1.40 kg DM/kg of BW) required to achieve ad libitum forage DM intake without concentrate supplementation (Inyang et al., 2010). Forage CP concentrations from day 0 to 77 were greater than the CP requirement of late-gestating beef cows (8.1% CP of DM; NASEM, 2016), whereas forage IVDOM concentrations from day 0 to 77 were below the TDN requirements of pregnant beef cows (53% TDN of DM; NASEM, 2016), explaining the BCS loss experienced by NOSUP cows from day 35 to 77. These responses were expected and in agreement with previous studies conducted in the same location and similar animals (Moriel et al., 2020b; Palmer et al., 2020, 2022a, b; Vedovatto et al., 2022).

Prepartum supplementation of protein and energy supplementation increased prepartum BCS of beef cows (Winterholler et al., 2012; Bohnert et al., 2013; Kennedy et al., 2019; Moriel et al., 2020b; Palmer et al., 2020, 2022a, b; Vedovatto et al., 2022), did not impact the percentage of pregnant beef cows in the subsequent breeding season (Stalker et al., 2007; Larson et al., 2009; Bohnert et al., 2013) but resulted in earlier calving compared to no prepartum supplementation of protein and energy (Palmer et al., 2020). Likewise, prepartum supplementation of DDG at 77 kg/cow from day 0 to 77, regardless of supplementation frequency, was sufficient to prevent or minimize prepartum BCS loss, leading to greater BCS during the first 30 d of the breeding season compared to NOSUP cows. Yet, the greater post-partum BCS of supplemented versus NOSUP cows was not sufficient to increase the percentage of pregnant cows on day 276 or decrease average calving date of first and second offspring. It is important to highlight that the present study was not designed to evaluate the reproductive performance of cows, which would require significantly greater number of cows and pasture replicates to achieve >80% power. However, similar outcomes were reported in our previous studies evaluating a wide variety of prepartum supplementation strategies for beef cows grazing warm-season forages in tropical/subtropical environments (Moriel et al., 2020b; Palmer et al., 2020, 2022a, b). A plausible explanation for the lack of treatment effects on cow reproduction is that NOSUP cows achieved acceptable pregnancy percentages likely because NOSUP cows calved at a BCS slightly below optimal levels (BCS = 5; 1 to 9 scale) and were able to maintain BCS from calving until the start of the breeding season.

Reduced frequency of concentrate supplementation either reduced (Cooke et al., 2008; Moriel et al., 2012; Artioli et al., 2015; Moriel et al., 2020a) or did not impact the growth performance of growing calves (Moriel et al., 2016b; Silva et al., 2018) but often decreased immune function of recently-weaned beef steers and heifers (Artioli et al., 2015; Moriel et al., 2016b; Silva et al., 2018) and reproductive performance of replacement beef heifers (Cooke et al., 2008; Moriel et al., 2012, 2020a). Discrepancies among those studies are probably related to differences on supplement composition, breed, gender, location, forage species and quality, and potential resulting interactions among these factors. In contrast, reduced frequency of concentrate supplementation did not impact the growth and reproductive performance of multiparous beef cows in previous studies (Klein et al., 2014; Moriel et al., 2016a) and herein, which supports our hypothesis. Reducing the frequency of concentrate supplementation from daily to three times weekly did not impact prepartum BCS loss of beef cows offered DDG supplementation at 0.4% of BW (DM basis) during mid- to late-gestation (Klein et al., 2014) and prepartum BCS change and post-partum reproduction of beef cows offered wet brewers grains DM supplementation at 0.5% of BW during the last 60 d of gestation (Moriel et al., 2016a). In the present study, decreasing the frequency of DDG supplementation did not impact BCS and BW and reproduction of multiparous beef cows, likely due to the capacity of beef cows to alter the metabolism of protein and energy (Cappellozza et al., 2015). These results also suggest that cows are more resilient to the detrimental effects of reduced frequency of concentrate supplementation on reproductive parameters compared to young, growing, replacement beef heifers. Hence, prepartum supplementation of protein and energy could be reduced to minimize feeding costs without detrimental effects to growth and reproduction of beef cows.

Plasma concentrations of glucose, IGF-1, and IGF-2 are positively correlated with energy and protein consumption (Sullivan et al., 2009; Cappellozza et al., 2014; Moriel et al., 2020b). However, the impacts of maternal supplementation of protein and energy on prepartum concentrations of glucose, IGF-1, and IGF-2 have been variable. In terms of maternal supplementation of protein and energy, prepartum plasma concentrations of IGF-1 and IGF-2, but not glucose, increased for growing beef heifers that received compared to cohorts that did not receive supplementation of sugarcane molasses and urea DM at 0.26% of BW (Moriel et al., 2020b). Prepartum plasma concentrations of glucose, but not IGF-1 and IGF-2, increased for multiparous beef cows supplemented with molasses and urea DM at 0.20% of BW compared to non-supplemented beef cows (Palmer et al., 2020). Prepartum DDG supplementation at either 0.20% or 0.40% of BW (DM basis) did not impact plasma concentrations of glucose and IGF-2 but increased plasma concentrations of IGF-1 during prepartum period compared to no supplementation of DDG (Palmer et al., 2022a). In terms of supplementation frequency, cows supplemented with wet brewers grains three times weekly during the last 60 d of gestation had greater plasma concentrations of glucose on days when concentrate was not provided compared to days when concentrate was provided (Moriel et al., 2016a), likely due to the time needed to synthesize and activate gluconeogenic enzymes (Cooke et al., 2008). Serum concentrations of NEFA on days when DDG was provided were greater for pregnant beef cows offered supplementation three times weekly or daily compared to non-supplemented beef cows; however, serum concentrations of NEFA on days when DDG supplementation was not provided were greater for non-supplemented cows compared to cows supplemented daily, whereas cows supplemented three times weekly were intermediate (Klein et al., 2014). Plasma concentrations of IGF-1 of beef heifers receiving daily supplementation of concentrate remained constant among blood collection days but were greater for heifers supplemented three times weekly on days when concentrate was provided compared to days when concentrate was not provided (Moriel et al., 2020a). Hence, these previous studies demonstrate that reducing the frequency of concentrate supplementation leads to variable effects on circulating concentrations of hormones and metabolites, and that the ranking of treatments in terms of circulating concentrations of metabolites and hormones is dynamic and dependent on timing of blood collection and type of metabolite and hormone being analyzed.

In the present study, overall plasma concentrations of glucose were greater for all cows that received DDG supplementation compared to NOSUP cows but were not affected by supplementation frequency. Likewise, plasma concentrations of IGF-1 on day 35 were greater for all supplemented cows compared to NOSUP cows, but the greatest increase occurred for 1× cows. To a lesser extent, plasma concentrations of IGF-1 on day 77 near calving were also greater for 1× cows compared to NOSUP cows and were intermediate for 3× and 7× cows, likely reflecting the greater DDG consumption on blood collection days compared to all remaining treatments. The discrepancy among the present study and those described above could be perhaps attributed to the differences in animal category, forage nutritive value, supplementation length, amount, and composition, and the timing at which supplementation was offered to cows during gestation. In addition, blood samples in the present study were only collected on days when concentrate supplementation was provided (Monday) due to the relatively high distance between pastures and the cattle processing facility preventing consecutive collections of blood samples. It is also possible that the lack of treatment effects (supplemented vs. non-supplemented as well as supplementation frequency) on plasma concentrations of IGF-2 and NEFA, and further differences on plasma concentrations of glucose among cows that received supplementation, could be attributed to the less intensive blood collection regimen compared to previous studies.

Transfer of immunoglobulins from maternal serum to colostrum in cattle begins approximately 28 d before calving and peaks near calving (Olson et al., 1981). Calf intake of IgG from colostrum and serum concentrations of IgG are positively correlated (Hopkins and Quigley, 1997). Maternal prepartum supplementation of protein and energy did not impact the concentration of IgG in the colostrum (Kennedy et al., 2019) and IgG concentration in plasma or serum of the offspring (Bohnert et al., 2013; Kennedy et al., 2019; Moriel et al., 2020b; Palmer et al., 2022a). Likewise, frequency of protein and energy supplementation did not affect circulating concentrations of IgG in the offspring (Klein et al., 2014; Moriel et al., 2016a). In the present study, serum concentrations of IgG in the first offspring were above levels required for adequate passive immune transfer (>16 mg/mL; Wittum and Perino, 1995) and were not impacted by maternal frequency of DDG supplementation during gestation.

Decreasing the frequency of maternal prepartum supplementation of wet brewers grains, from daily to three times weekly, increased the plasma concentrations of haptoglobin in the offspring at the time of birth (Moriel et al., 2016a) to levels above those considered as an indicator of physiological stress and inflammatory response (≥0.11 mg/mL; Tourlomoussis et al., 2004). In the present study, plasma concentrations of haptoglobin were above 0.11 mg/mL, regardless of treatment, indicating that calves experienced an inflammatory response following birth. However, contrary to our previous study (Moriel et al. (2016a) and to our hypothesis, plasma concentrations of haptoglobin at birth of the first offspring was not impacted by previous maternal frequency of supplementation.

Fetal growth depends on nutrients availability during gestation (Bauer et al., 1998) with glucose and amino acids being vital for fetal growth (Bell, 2005). Glucose availability to the growing fetus is regulated by maternal circulating concentrations of glucose (Baumann et al., 2002), whereas plasma concentrations of IGF-1 and IGF-2 are synthesized and regulated by the placenta, maternal and fetal tissues (Gicquel and Le Bouc, 2006). Therefore, maternal supplementation of protein and energy during gestation may influence the offspring BW at birth by altering nutrient availability and glucose transfer to the fetus via IGF system regulation (Sferruzzi-Perri et al., 2006). Nonetheless, variable results on offspring birth BW have been reported with nearly half of the studies observing that prepartum supplementation of protein and energy had no effects or increased offspring birth BW by on average 3.2 kg (Moriel et al., 2021). In the present study, birth BW of the first offspring tended to be greater for calves born from 3× and 7× cows (and numerically greater for 1× calves) compared to NOSUP cows and no effects of supplementation frequency were observed for offspring birth BW, corroborating with previous studies (Klein et al., 2014; Moriel et al., 2016a). These results likely reflect the differences observed for cow prepartum BCS change and plasma concentrations of maternal glucose and IGF-1. It is also possible that the plasma concentrations of IGF-1 of 1× cows peaked immediately after DDG supplementation but returned and remained at baseline levels until subsequent DDG supplementation was provided, limiting IGF-1 actions and glucose uptake by the fetus compared to 3× and 7× cows which received a more constant supply of nutrients during the week.

Inconsistent results were also observed for calf preweaning growth, with nearly half of the studies reporting either no effects or increased calf BW at weaning following maternal supplementation of protein and energy during gestation (Moriel et al., 2021). Prepartum supplementation of DDG increased offspring BW at the time of weaning (Bohnert et al., 2013; Kennedy et al., 2019; Palmer et al., 2022a) compared to no prepartum supplementation of DDG. Corroborating with previous studies, calves born from cows offered DDG supplementation, regardless of supplementation frequency, had greater BW at weaning compared to NOSUP calves. Perhaps the same rationale described above for the effects of maternal supplemental intake of protein and energy on fetal nutrient availability and regulation (i.e., fetal uptake of glucose via IGF-1 system) and resulting effects on offspring BW at birth could also be applied for the observed differences on offspring preweaning growth. Calf BW at weaning did not differ between 1× and 3× calves but were greater for 7× calves, which partially agrees with our hypothesis and supports the rationale that a more consistent supply of nutrients during gestation improves nutrient availability and use by the fetus and potentially in utero programming of fetal growth, optimizing offspring preweaning BW gain. In agreement with this rationale, maternal supplementation of DDG for only the first half of the third trimester of gestation enhanced calf preweaning BW compared to calves born from non-supplemented beef cows but to a much lesser magnitude compared with maternal supplementation of DDG during the entire third trimester of gestation (6 vs. 14 kg of added offspring BW at weaning, respectively; Palmer et al., 2022a).

## CONCLUSION

In summary, decreasing the frequency of maternal supplementation of protein and energy (via high-DM concentrate) during third trimester of gestation did not affect pre- and post-partum BCS and reproductive performance of beef cows but altered their plasma concentrations of glucose and IGF-1 during pregnancy. Contrary, decreasing the frequency of maternal supplementation of protein and energy during third trimester of gestation reduced preweaning growth performance of the beef offspring. Combined, these data indicate that a more consistent supply of supplemental protein and energy during third trimester of gestation does not improve maternal performance but was required to optimize calf growth performance during the preweaning stage
